# Fish Autophagy Protein 5 Exerts Negative Regulation on Antiviral Immune Response Against Iridovirus and Nodavirus

**DOI:** 10.3389/fimmu.2019.00517

**Published:** 2019-03-19

**Authors:** Chen Li, Jiaxin Liu, Xin Zhang, Shina Wei, Xiaohong Huang, Youhua Huang, Jingguang Wei, Qiwei Qin

**Affiliations:** ^1^Joint Laboratory of Guangdong Province and Hong Kong Region on Marine Bioresource Conservation and Exploitation, College of Marine Sciences, South China Agricultural University, Guangzhou, China; ^2^Laboratory for Marine Biology and Biotechnology, Qingdao National Laboratory for Marine Science and Technology, Qingdao, China

**Keywords:** grouper, Atg5, SGIV, RGNNV, interferon

## Abstract

Autophagy is an important biological activity that maintains homeostasis in eukaryotic cells. However, little is known about the functions of fish autophagy-related genes (Atgs). In this study, we cloned and characterized Atg5, a key gene in the autophagy gene superfamily, from orange-spotted grouper (*Epinephelus coioides*) (EcAtg5). EcAtg5 encoded a 275-amino acid protein that shared 94 and 81% identity to seabass (*Lates calcarifer*) and humans (*Homo sapiens*), respectively. The transcription level of EcAtg5 was significantly increased in cells infected with red-spotted grouper nervous necrosis virus (RGNNV). In cells infected with Singapore grouper iridovirus (SGIV), EcAtg5 expression declined during the early stage of infection and increased in the late stage. Fluorescence microscopy revealed that EcAtg5 mainly localized with a dot-like pattern in the cytoplasm of grouper cells. Overexpression of EcAtg5 significantly increased the replication of RGNNV and SGIV at different levels of detection, as indicated by increased severity of the cytopathic effect, transcription levels of viral genes, and levels of viral proteins. Knockdown of EcAtg5 decreased the replication of RGNNV and SGIV. Further studies showed that overexpression EcAtg5 activated autophagy, decreased expression levels of interferon related cytokines or effectors and pro-inflammatory factors, and inhibited the activation of nuclear factor κB, IFN-sensitive response element, and IFNs. In addition, ectopic expression of EcAtg5 affected cell cycle progression by hindering the G1/S transition. Taken together, our results demonstrated that fish Atg5 exerted a crucial role in virus replication by promoting autophagy, down-regulating antiviral IFN responses, and affecting the cell cycle.

## Introduction

Autophagy is a conserved cell biological pathway that delivers cytoplasmic components to lysosomes for degradation and elimination of useless or harmful substrates to maintain cell homeostasis in all eukaryotic cells (1). This fundamental process involves formation of double membrane autophagosomes, which fuse with lysosomes to degrade the sequestered cargo (2, 3). Viruses depend on the host cell's machinery to replicate the genome and generate progeny virus particles. Autophagy, as a cell steward, has been reported to play an important role in virus replication. Many studies have reported that viruses cause accumulation of autophagosomes and exploit these membrane structures as “virus factories” (4). In addition, several studies have confirmed that autophagy interacts with the innate antiviral immune response (5–7). Autophagy can amplify the innate immune response that is mediated by nucleic acid-sensing Toll-like receptors and enhance delivery of cytosolic pathogen-associated molecular patterns (8). Additionally, some autophagy factors can downregulate RIG-I (retinoic acid-inducible gene I)-like receptors and type I interferon (IFN) signaling and suppress inflammasome activation (9, 10).

The process of autophagosome formation is regulated by several autophagy-related genes (Atgs) (11). In the autophagy gene superfamily, Atg5 is a key gene that plays an important role in early autophagosome formation. Enhanced or reduced Atg5 levels affect the occurrence and alteration of autophagy pathways. On one hand, Atg5 protein can conjugate to Atg12 to form a complex with the multimeric protein Atg16, and the Atg12-Atg5-Atg16 complex facilitates extension of the autophagosome (12, 13). On the other hand, the combination of Atg5 complex and autophagic vesicle membrane can promote recruitment of LC3 (Atg8) to autophagic vesicles (14). Atg5 is involved in various physiological and pathological processes. In lipid metabolism, silencing or knocking out Atg5 can lead to lipid deposition. Atg5 also plays a role in regulating IFN immune and inflammation responses (7).

The grouper (*Epinephelus* spp.) is a well-known mariculture species that is widely distributed in South China and Southeast Asia. In 2016, the scale of grouper breeding in China was 108,319 t, which was 8.31% higher than that in 2015. However, outbreaks of viral diseases have caused heavy economic losses in the grouper aquaculture industry. Two representative pathogens are Singapore grouper iridovirus (SGIV) and red-spotted grouper nervous necrosis virus (RGNNV) (15, 16). Current research on the prevention and control of viral diseases in grouper is mainly focused on exploration of the anti-virus immune network and key immune genes. Although numerous immune regulatory molecules have been found to play vital roles in the grouper antiviral response (17–21), the roles of Atgs in the replication of SGIV or RGNNV have not been reported. In other studies of aquatic viruses, proliferation of SVCV was significantly reduced in Beclin-1(Atg6) and LC3(Atg8)-depleted endothelial progenitor cells. However, references to Atg5 in aquatic animal viruses are limited (22).

In this study, we cloned a key autophagy related gene (Atg5) from orange-spotted grouper (*E. coioides*) (EcAtg5) and investigated the roles of EcAtg5 in autophagy, innate immunity, and cell cycle. Our results provide new insights into the roles of fish Atg5 in virus infection.

## Materials and Methods

### Cloning of EcAtg5 and Bioinformatic Analysis

Based on several expressed sequence tag sequences of EcAtg5 from the grouper spleen transcriptome (23), primers (Table 1) were designed to amplify the full-length open reading frame (ORF) of EcAtg5. Identity analysis between EcAtg5 and other Atg5 sequences was performed using BLASTP searches of the NCBI database. Amino acid alignments were conducted using MEGA5.0 software and edited with the GeneDoc program. The phylogenetic analysis was carried out using the boot-strapped neighbor joining method in ClustalX 2.1 software.

**Table 1:** Primers used in this study.

**Name**	**Sequence (5′-3′)**
EcAtg5-F	ATGGCAGATGACAAGG
EcAtg5-R	TCAGTCAGTGGGGACGG
C1-EcAtg5-F	GAAGATCTATGGCAGATGACAAGGAT
C1-EcAtg5-R	GGGGTACCGTCAGTGGGGACGGGGATAA
HA-EcAtg5-F	GGGGTA CCATGGCAGATGACAAGG
HA-EcAtg5-R	CGGAATTCTTCAGTCAGTGGGGACGG
EcAtg5-RT-F	CCACTGAGGAGGGAGGCTT
EcAtg5-RT-R	CAGATGAAACAGGGCGAAA
Actin-RT-F	TACGAGCTGCCTGACGGACA
Actin-RT-R	GGCTGTGATCTCCTTCTGCA
MCP-RT-F	GCACGCTTCTCTCACCTTCA
MCP-RT-R	AACGGCAACGGGAGCACTA
ICP-18-RT-F	ATCGGATCTACGTGGTTGG
ICP-18-RT-R	CCGTCGTCGGTGTCTATTC
VP19-RT-F	TCCAAGGGAGAAACTGTAAG
VP19-RT-R	GGGGTAAGCGTGAAGACT
LITAF-RT-F	GATGCTGCCGTGTGAACTG
LITAF-RT-R	GCACATCCTTGGTGGTGTTG
RdRp-RT-F	GTGTCCGGAGAGGTTAAGGATG
RdRp-RT-R	CTTGAATTGATCAACGGTGAACA
CP-RT-F	CAACTGACAACGATCACACCTTC
CP-RT-R	CAATCGAACACTCCAGCGACA
EcIRF3-RT-F	ATGGTTTAGATGTGGGGGTGTCGGG
EcIRF3-RT-R	GAGGCAGAAGAACAGGGAGCACGGA
EcIRF7-RT-F	CAACACCGGATACAACCAAG
EcIRF7-RT-R	GTTCTCAACTGCTACATAGGGC
EcISG15-RT-F	CCTATGACATCAAAGCTGACGAGAC
EcISG15-RT-R	GTGCTGTTGGCAGTGACGTTGTAGT
EcMDA5-RT-F	ACCTGGCTCTCAGAATTACGAACA
EcMDA5-RT-R	TCTGCTCCTGGTGGTATTCGTTC
EcMyD88-RT-F	AGCTGGAGCAGACGGAGTG
EcMyD88-RT-R	GAGGCTGAGAGCAAACTTGGTC
EcMXI-RT-F	CGAAAGTACCGTGGACGAGAA
EcMXI-RT-R	TGTTTGATCTGCTCCTTGACCAT
EcLGP2-RT-F	TGGTGGTACGCTATGGACTGC
EcLGP2-RT-R	TTGTAGCTCAGTTATCTTTGTGCGA
EcIFP35-RT-F	TTCAGATGAGGAGTTCTCTCTTGTG
EcIFP35-RT-R	TCATATCGGTGCTCGTCTACTTTCA
EcTRAF6-RT-F	CCCTATCTGCCTTATGGCTTTGA
EcTRAF6-RT-R	ACAGCGGACAGTTAGCGAGAGTAT
EcTNFα-RT-F	GTGTCCTGCTGTTTGCTTGGTA
EcTNFα-RT-R	CAGTGTCCGACTTGATTAGTGCTT
EcIL6-RT-F	CTCTACACTCAACGCGTACATGC
EcIL6-RT-R	TCATCTTCAAACTGCTTTTCGTG
EcIL-1β-RT-F	AACCTCATCATCGCCACACA
EcIL-1β-RT-R	AGTTGCCTCACAACCGAACAC
EcIL8-RT-F	GCCGTCAGTGAAGGGAGTCTAG
EcIL8-RT-R	ATCGCAGTGGGAGTTTGCA

### Tissue Distribution Analysis of EcAtg5

Orange-spotted groupers (30–40 g) used in this study were purchased from a local farm in Hainan Province and kept in a laboratory recirculating seawater system as described previously (20). The relative expression level of EcAtg5 was examined using quantitative real-time PCR (qRT-PCR) in selected tissues, including liver, spleen, head kidney, kidney, heart, intestine, brain, gill, stomach, muscle, fin, and skin. All tissues were collected from three fish.

### Cells and Virus

A grouper spleen (GS) cell line was established in our lab (24), and cells were propagated and maintained at 28°C in Leibovitz's L-15 medium (Gibco, USA) supplemented with 10% fetal bovine serum (Gibco, USA). SGIV and RGNNV were isolated in our laboratory and propagated in GS cells with titer of 10^5^ TCID_50_/ml as described previously (15, 16).

### Plasmid Construction

To clarify the molecular function of EcAtg5 *in vitro*, EcAtg5 was subcloned into the vectors pEGFP-C1 and pcDNA3.1-3 × HA using the primers listed in Table 1. All recombinant plasmids were confirmed by DNA sequencing.

### siRNA-Mediated EcAtg5 Knockdown

GS cells were transfected with EcAtg5 siRNA (siEcAtg5:5′-GAAAGAGAUGUACCCUGCUGCUUUA-3′) or same volume negative control for 24 h, and then infected with SGIV or RGNNV for 12, 24, and 36 h. At the end of each incubation period, the total RNA or protein of cells were extracted for detection.

### Cellular Localization Analysis

GS cells were seeded onto cover slips (10 × 10 mm) in 24-well plates. After allowing the cells to adhere for 24 h, pEGFP-C1 and pEGFP-EcAtg5 plasmids were transfected into GS cells using the transfection reagent Lipofectamine 2000 (Invitrogen, Carlsbad, CA, USA). At 24 h post-transfection, cells on the cover slips were washed with phosphate buffered saline (PBS), fixed with 4% paraformaldehyde for 1 h at room temperature, and then stained with 4,6-diamidino-2-pheny-lindole (DAPI) for 10 min. Finally, cells were observed under a fluorescence microscope (Leica, Wetzlar, Germany).

### RNA Extraction and Gene Expression Analysis

Total RNA isolation was performed using the SV Total RNA Isolation System (Promega, USA) according the manufacturer's instructions, and reverse transcription was carried out using ReverTra Ace (Toyobo, Osaka, Japan). qRT-PCR was performed in an ABI Quant studio 5 device (Applied Biosystems, Carlsbad, CA, USA). The expression levels of viral genes and host immune genes were detected. The relative expression ratio of the selected gene vs. β-actin (reference gene) was calculated using the 2^−ΔΔ^CT method. Reactions of SYBR Green were performed in a 10 μl volume containing 5 μl of 2 × SYBR® Premix Ex Taq™, 0.3 μl of each forward and reverse primer (10 μM), 3.4 μl of water, and 1 μl of cDNA. All experiments were performed in triplicate, and the cycling parameters were chosen according to the manufacturer's instructions.

### Western Blot Analysis to Measure Protein Levels

Cells were collected and lysed in RIPA buffer. Proteins were separated by 12% SDS-PAGE and transferred onto Immobilon-P polyvinylidene difluoride membranes (Millipore, Temecula, CA, USA). Blots were incubated with the indicated primary antibody: anti-HA (1:1,000 dilution), anti-β-actin (1:1,000 dilution), anti-ATG5 (1:500 dilution), anti-LC3 (1:1,000 dilution), anti-SGIV major capsid protein (MCP) (1:1,000 dilution), anti-RGNNV capsid protein (CP) (1:1,000 dilution). Subsequently they were incubated with horseradish peroxidase (HRP)-conjugated goat-anti-rabbit IgG (1:5,000 dilution). Mouse monoclonal anti-HA antibody was purchased from Sigma (USA). Mouse monoclonal anti-β-actin antibody and rabbit polyclonal ATG5 antibody were purchased from Proteintech (Rosemont, IL, USA). Rabbit monoclonal anti-LC3 antibody was purchased from Abcam (USA). HRP-conjugated goat anti-rabbit and anti-mouse antibodies were purchased from KPL (USA). The polyclonal anti-MCP antibody of SGIV and the polyclonal anti-CP antibody of RGNNV were prepared in our lab. Immunoreactive proteins were visualized using an Enhanced HRP-DAB Chromogenic Substrate Kit (Tiangen, Beijing, China).

### Dual-Luciferase Reporter Assays

To evaluate the promoter activity regulated by EcAtg5, luciferase reporter plasmids, including interferon-sensitive response element (ISRE)-Luc, IFN3-Luc, and nuclear factor (NF)-κB (Clontech, USA), were used for co-transfection. Briefly, GS cells were transiently transfected with the luciferase plasmids together with the indicated EcAtg5 expression vectors using Lipofectamine 2000 reagent. The pRL-SV40 Renilla luciferase vector was used as an internal control. Luciferase activity of total cell lysates was measured using a luciferase reporter assay system (Promega).

### Flow Cytometry Analysis of the Cell Cycle

To evaluate the role of EcAtg5 in cell cycle progression, GS cells were transfected with pcDNA3.1-3 × HA-EcAtg5 or the empty vector. At 36 h post-transfection, cells were harvested and fixed in 70% ice-cold ethanol overnight at −30°C. Cells then were washed with PBS and centrifuged for subsequent incubation in PBS containing 50 mg/mL of propidium iodide (PI) and 100 mg/mL of RNaseA for 30 min. The PI fluorescence was measured with a Beckman Coulter flow cytometer (Brea, CA, USA), and 10,000 cells were analyzed for each sample. The data were analyzed using ModFit LT 4.1 software.

### Statistical Analysis

Statistical analysis was performed using SPSS Version 13. One-way ANOVA was used to evaluate the variability between treatment groups (^*^p < 0.05, ^**^p < 0.01).

## Results

### Characterization of EcAtg5

**Figure 1:**
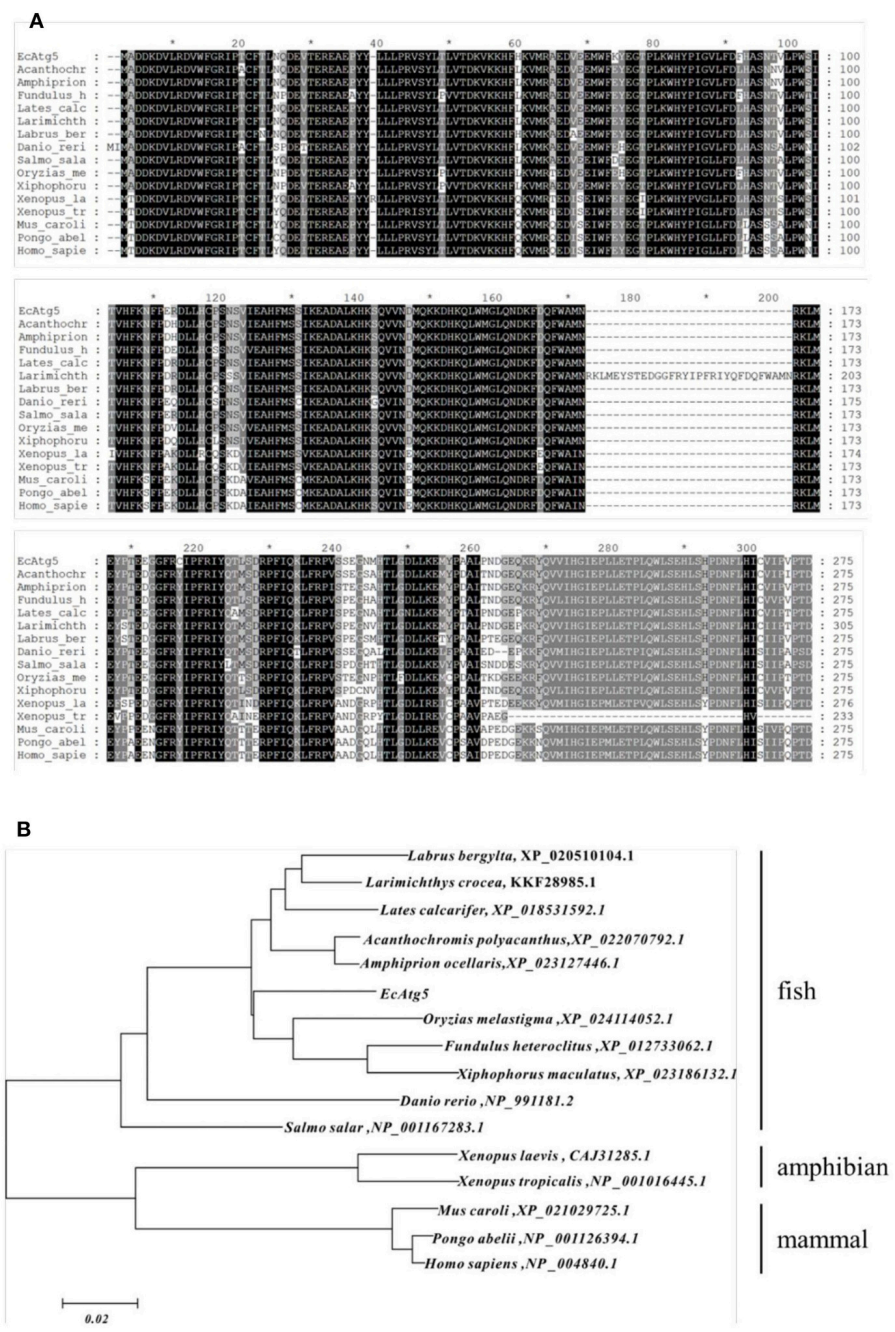
**(A)** Multiple sequence alignment of EcAtg5 and other Atg5 homologs from different species. Amino acid alignments were performed using MEGA5.0 software and edited with the GeneDoc program. **(B)** Phylogenetic analysis of EcAtg5. All sequences of Atg5 homologs from different species were obtained from the NCBI database. The phylogenetic analysis was carried out using the boot-strapped neighbor joining method in ClustalX 2.1 software.

The full-length ORF of EcAtg5 was obtained using PCR amplification. Sequence analysis indicated that EcAtg5 encoded a 275-amino acid protein that shared 94% and 81% identity to seabass (*Lates calcarifer*) and humans (*Homo sapiens*), respectively (Figure 1A). Phylogenetic analysis indicated that EcAtg5 was closely related to the fish subgroup, followed by amphibians, birds, and mammals (Figure 1B).

### Expression Patterns of EcAtg5

**Figure 2:**
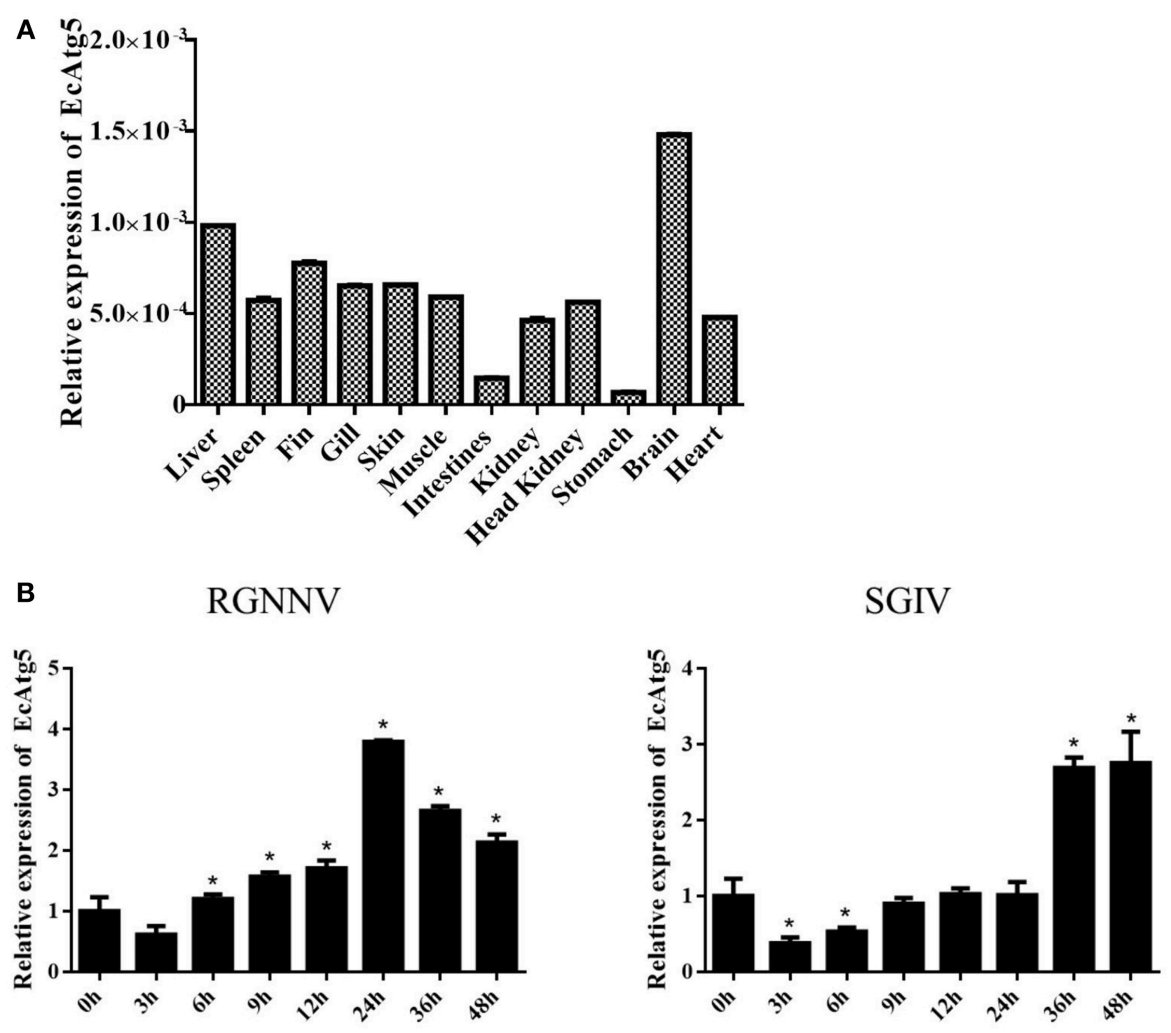
Expression profiles of EcAtg5. **(A)** Tissue distribution patterns of EcAtg5 in healthy grouper. Data were expressed as a ratio to EcAtg5 mRNA expression in stomach. **(B)** Expression changes of EcAtg5 in response to challenge with RGNNV/SGIV for the indicated length of time, at which time cells were collected for RNA extraction and qRT-PCR analysis. One-way ANOVA was used to evaluate the variability between treatment groups (*p < 0.05).

To analyze the tissue distribution, qRT-PCR was conducted in different tissues of healthy juvenile orange-spotted grouper. EcAtg5 was constitutively expressed in all the analyzed tissues in healthy grouper, and it was relatively high mRNA levels in the brain, liver, and fin (Figure 2A). To analyze the gene expression profiles in response to different viral infections, the transcription levels of EcAtg5 were examined in RGNNV or SGIV infected cells. The transcription levels of EcAtg5 were significantly increased in RGNNV infected cells. In SGIV infected cells, the expression levels of EcAtg5 first decreased within 24 h post-injection and then increased after 36 h (Figure 2B).

### EcAtg5 Encodes a Cytoplasmic Protein

**Figure 3:**
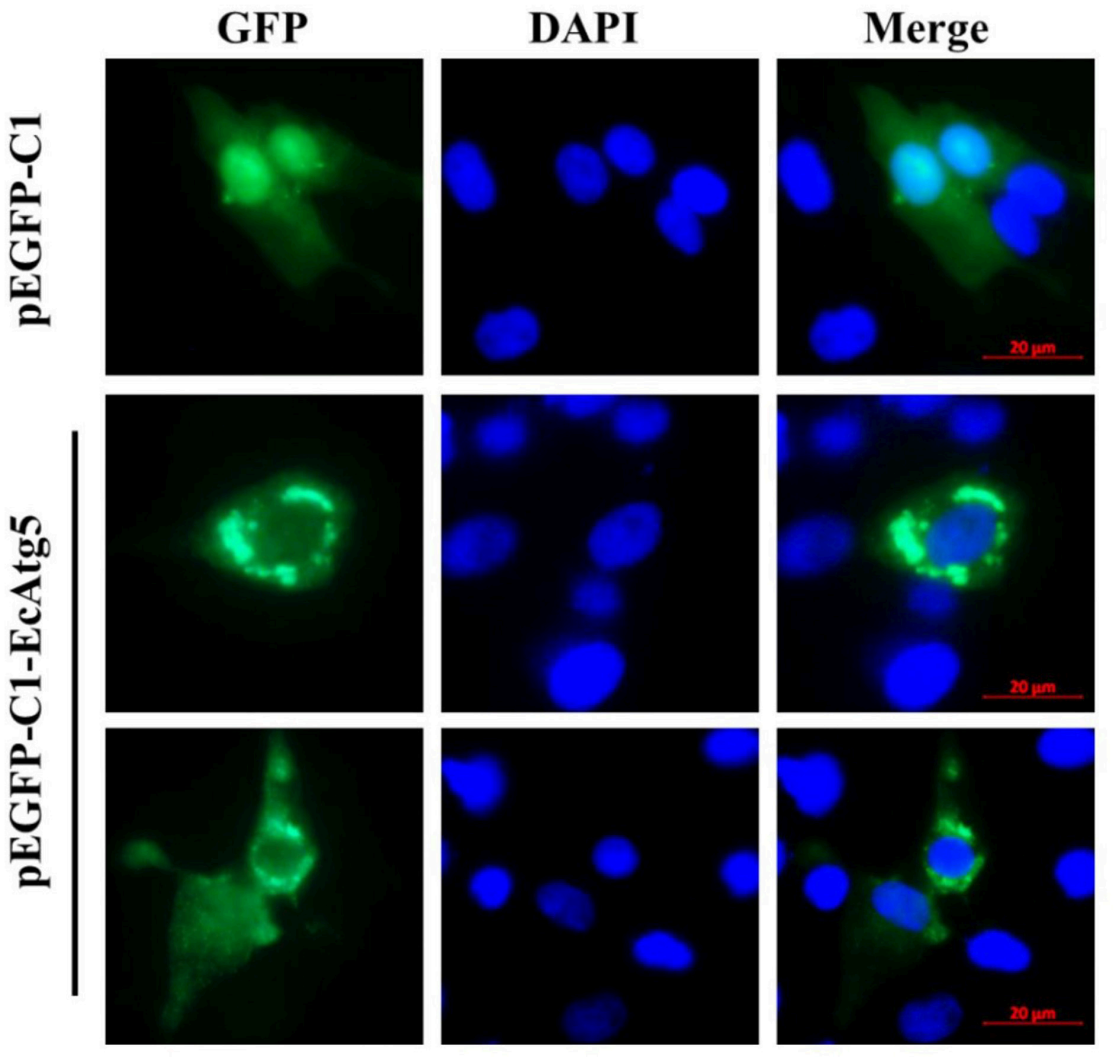
Subcellular localization of EcAtg5 in grouper cells. GS cells were transfected with pEGFP-C1 or pEGFP-EcAtg5. After 24 h, fixed cells were stained with DAPI and imaged by fluorescence microscopy.

To demonstrate the subcellular localization of EcAtg5, pEGFP-EcAtg5 was transfected into grouper cells, and fluorescence was observed under fluorescence microscopy. Green fluorescence was observed in the cytoplasm in EcAtg5 transfected grouper cells, and most of these cells exhibited fluorescence aggregation (Figure 3). In pEGFP-C1 transfected cells, fluorescence was distributed both the cytoplasm and nucleus. The results showed that EcAtg5 was a cytoplasmic protein.

### EcAtg5 Triggered Autophagy in GS Cells

**Figure 4:**
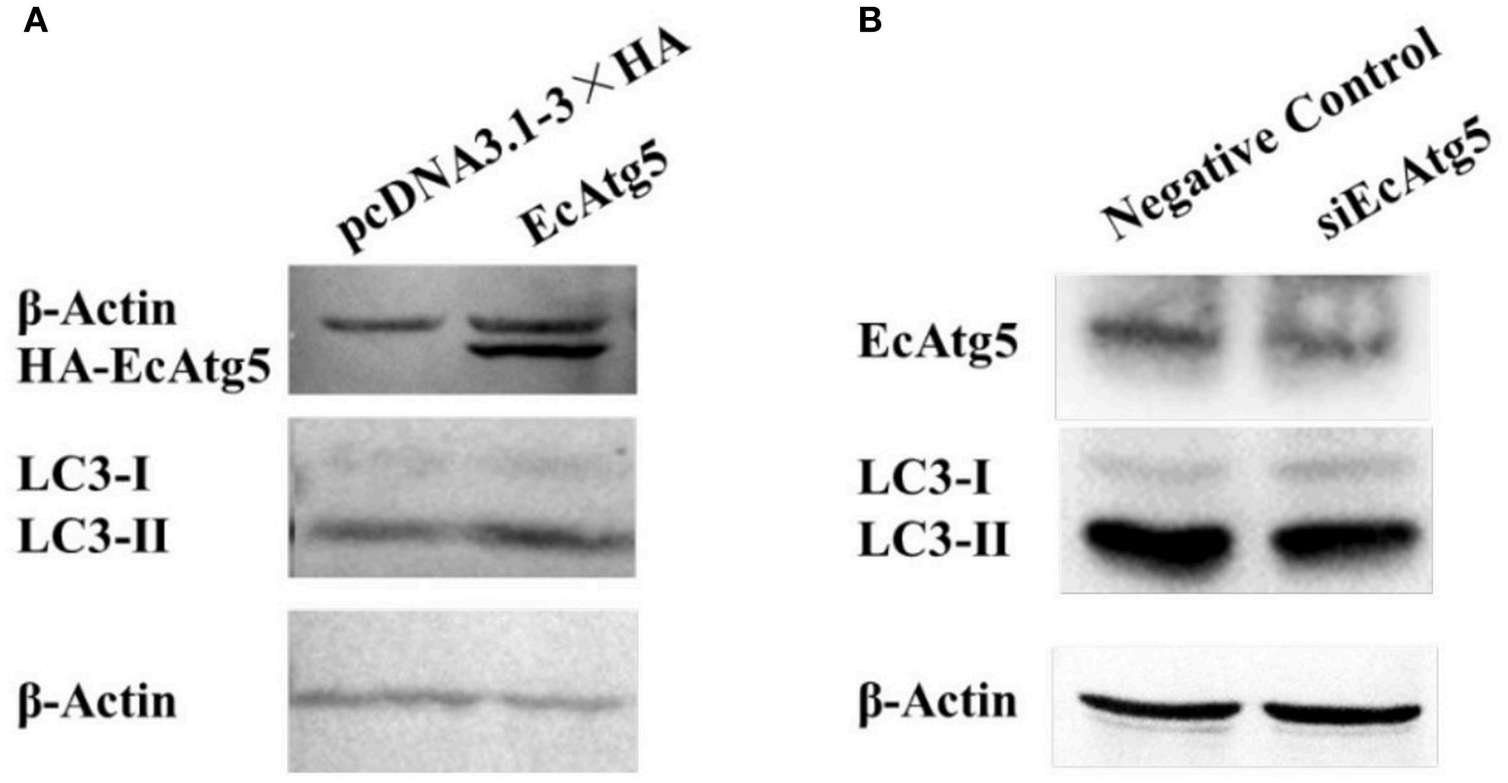
Effects of EcAtg5 on autophagy marker LC3. **(A)** EcAtg5 overexpression increased the level of LC3-II. pcDNA3.1-3 × HA-EcAtg5 and the empty vector were transfected into GS cells, respectively. After 24 h, cells were harvested for immunoblotting with an anti-HA antibody or anti-LC3 antibody, and β-actin was used as the internal control. **(B)** Knockdown of EcAtg5 reduced the level of LC3-II. Cells were transfected with siEcAtg5, then the level of EcAtg5 or LC3 was detected by Western blot, and β-actin was used as the internal control.

To clarify the function of EcAtg5, the eukaryotic expression vector of pcDNA3.1-3 × HA-EcAtg5 was constructed, and the recombinant plasmid successfully expressed HA-EcAtg5 protein after being transfected into GS cells (Figure 4A). On the contrary, EcAtg5 protein level was decreased after siRNA silencing (Figure 4B).

Autophagy is characterized by the formation of autophagosomes. Conjugation of the essential LC3 to phosphatidylethanolamine is required for autophagosome biogenesis. Therefore, LC3 lipidation is used as a faithful marker of autophagy activation (22, 25). To assess whether EcAtg5 overexpression affected GS autophagy, we investigated the level of LC3 lipidation in cells overexpressing or silencing EcAtg5. The LC3-II (the lipidated form) level was higher in cells transfected with EcAtg5 compared with control cells (Figure 4A), and EcAtg5 knockdown reduced the LC3-II level (Figure 4B), which suggests that EcAtg5 might activate autophagy by promoting LC3 lipidation in GS cells.

### EcAtg5 Increased SGIV and RGNNV Replication

**Figure 5:**
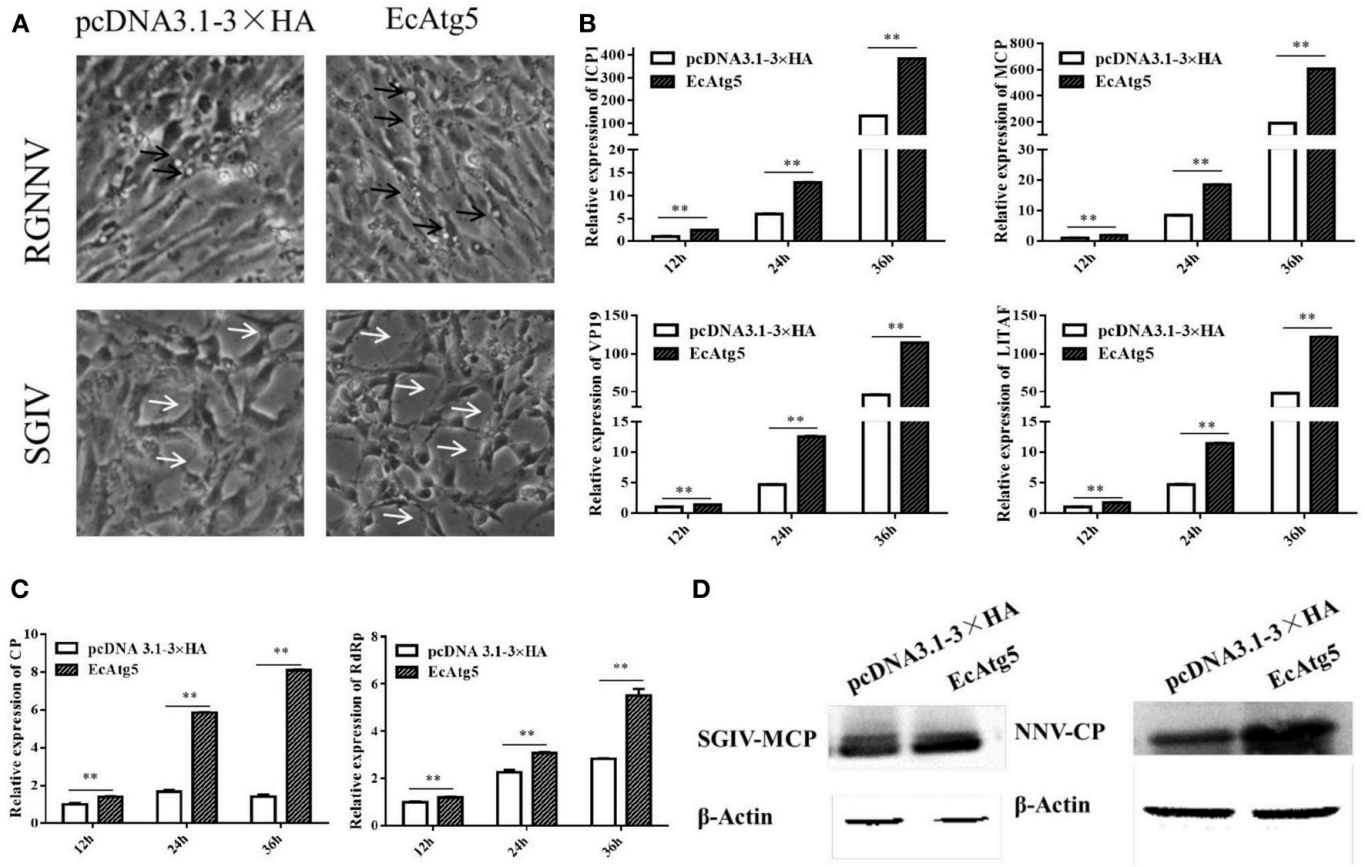
Effect of EcAtg5 overexpression on virus replication. **(A)** CPE were induced by SGIV or RGNNV after transfection with EcAtg5. The black arrows indicate the vacuoles evoked by RGNNV infection, and the white arrows show the CPE induced by SGIV infection. **(B)** EcAtg5 overexpression increased SGIV gene transcription. Expression levels of MCP, ICP18, VP19, and LITAF were determined using qRT-PCR. **(C)** EcAtg5 overexpression increased RGNNV gene transcription. Expression levels of CP and RdRp were determined using qRT-PCR. One-way ANOVA was used to evaluate the variability between treatment groups (**p < 0.01). **(D)** Virus protein level after transfection with EcAtg5. The level of SGIV-MCP or RGNNV-CP was detected by Western blot, and β-actin was used as the internal control.

To clarify the effects of EcAtg5 overexpression on virus infection, EcAtg5 transfected cells were infected with SGIV or RGNNV, and then viral replication was investigated. Severity of the cytopathic effect (CPE) induced by SGIV infection evoked at 24 h (Figure 5A). The amount and severity of vacuoles induced by RGNNV infection also increased in EcAtg5 overexpressing cells compared to the empty vector transfected cells. At the transcription level, the expression of SGIV MCP, ICP18, VP19, and LITAF increased in EcAtg5 overexpressing cells after SGIV infection (Figure 5B). The transcription of RGNNV CP and RdRp genes also increased compared with control cells after RGNNV infection (Figure 5C). Consistently, ectopic expression of EcAtg5 also increased the protein levels of SGIV MCP and RGNNV CP (Figure 5D).

**Figure 6:**
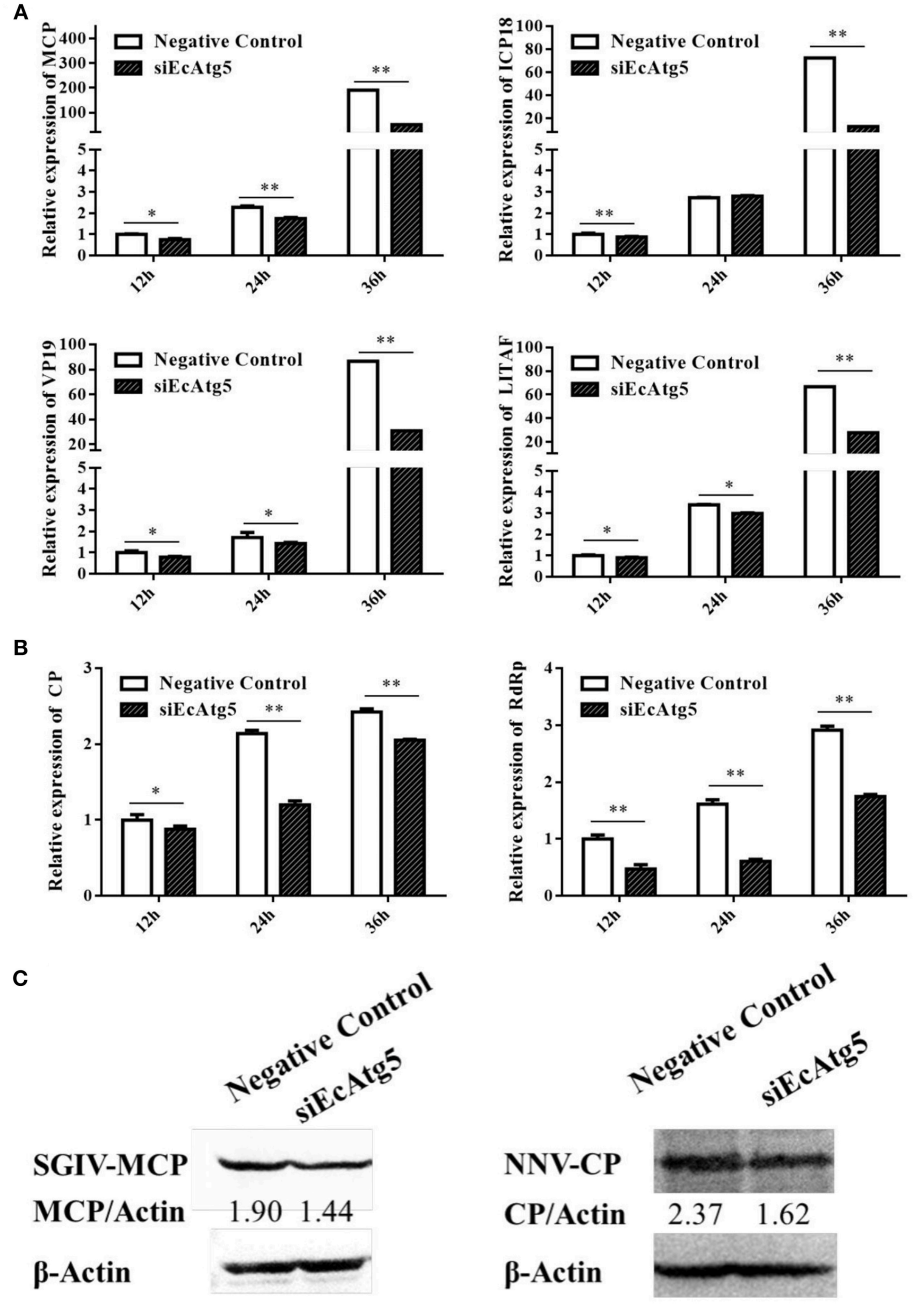
Effect of EcAtg5 knockdown on virus replication. **(A)** Knockdown of EcAtg5 decreased SGIV gene transcription including MCP, ICP18, VP19, and LITAF. **(B)** Knockdown of EcAtg5 decreased RGNNV gene transcription including CP and RdRp. One-way ANOVA was used to evaluate the variability between treatment groups (*p < 0.05, **p < 0.01). **(C)** Virus protein level after transfection with siEcAtg5. The level of SGIV-MCP or RGNNV-CP was detected by Western blot, and β-actin was used as the internal control. Band intensity was calculated using Quantity-one software and ratios of MCPor CP/β-actin was assessed (*p* < 0.05).

Meanwhile, the effects of silencing EcAtg5 on SGIV and RGNNV replication were also studied. Quantitative analysis results showed that the transcription level of SGIV MCP, ICP18, VP19, and LITAF decreased after EcAtg5 knockdown (Figure 6A). The transcription of RGNNV CP and RdRp genes also decreased compared with negative control cells (Figure 6B). Consistently, EcAtg5 knockdown decreased the protein levels of SGIV MCP and RGNNV CP in GS cells (Figure 6C). Together, EcAtg5 was speculated to promote SGIV and RGNNV replication in GS cells.

### Overexpression of EcAtg5 Decreased the Interferon Immune Response and Pro-Inflammatory Cytokines

**Figure 7:**
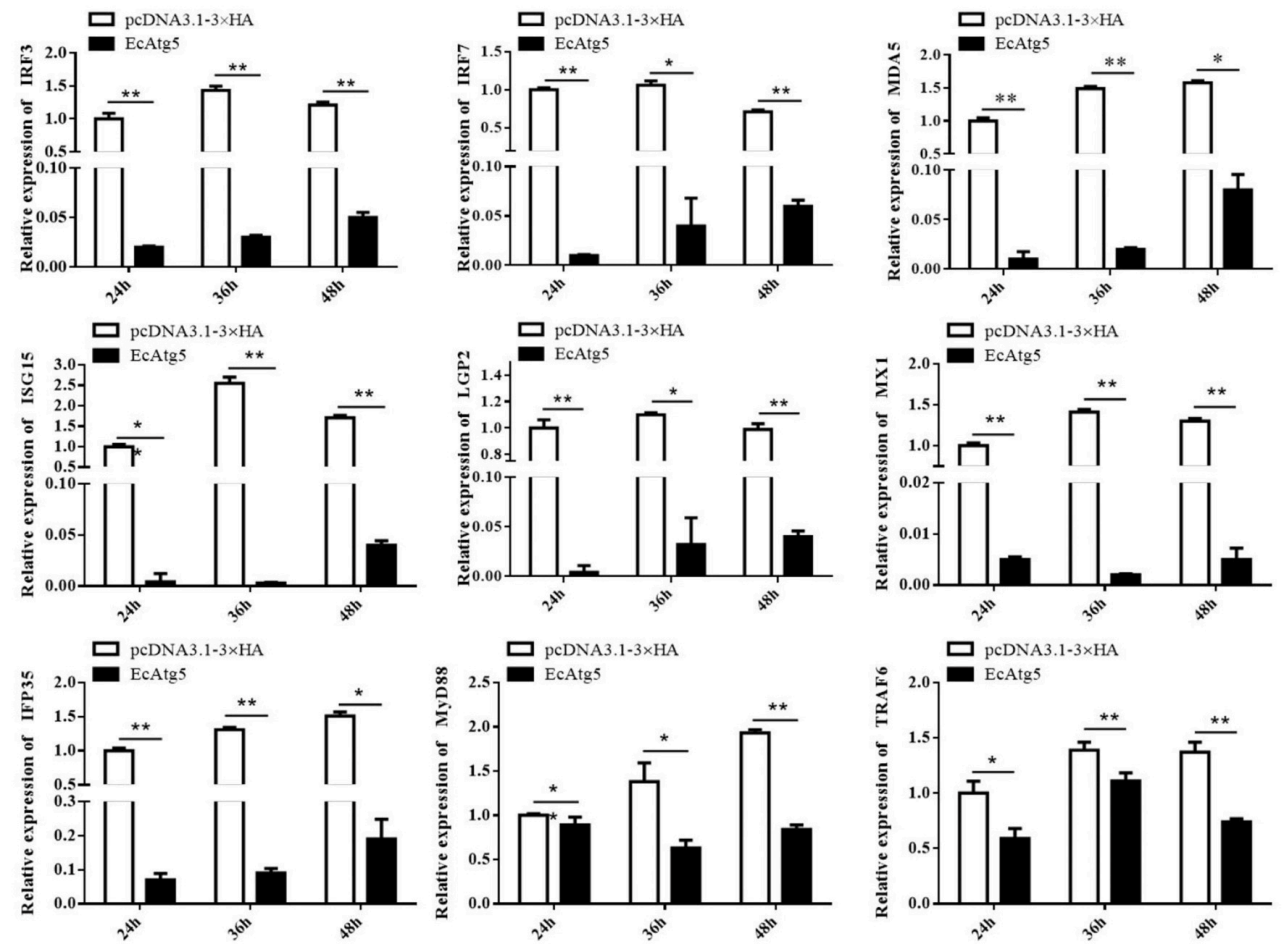
EcAtg5 overexpression decreased expression of interferon related cytokines or effectors. GS cells were transfected with EcAtg5, then cells were harvested at the indicated time. Expression levels of host IFN associated genes were determined using qRT-PCR. One-way ANOVA was used to evaluate the variability between treatment groups (*p < 0.05, **p < 0.01).

**Figure 8:**
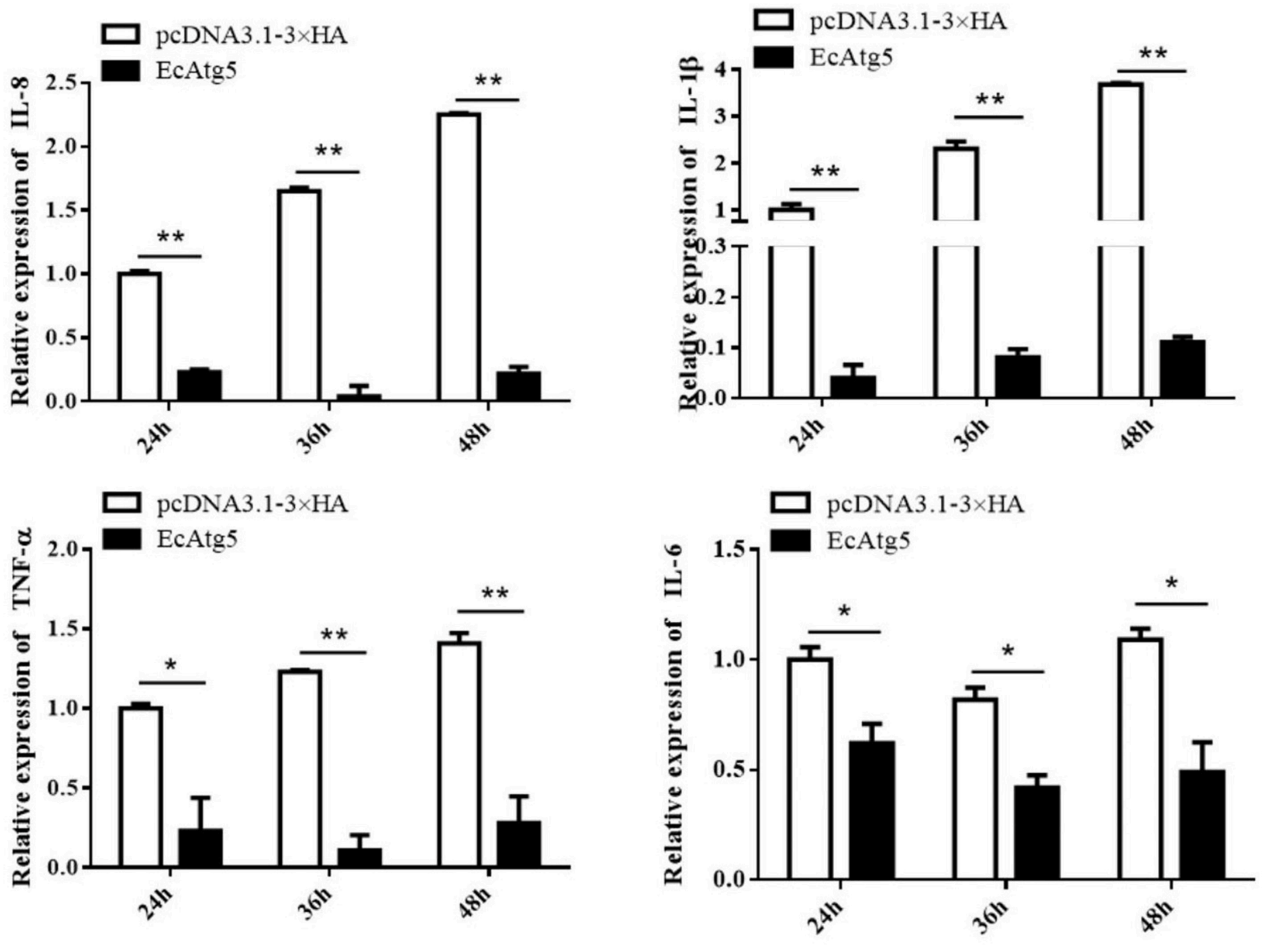
Overexpression of EcAtg5 decreased the transcription of proinflammatory factors. GS cells were transfected with EcAtg5, then cells were harvested at the indicated time. Expression levels of proinflammatory factors were determined using qRT-PCR. One-way ANOVA was used to evaluate the variability between treatment groups (*p < 0.05, **p < 0.01).

To explore the potential mechanism involved in the action of EcAtg5 in fish virus infections, the roles of EcAtg5 on the host interferon immune and inflammation response were evaluated. The pcDNA3.1-3 × HA-EcAtg5 and the empty vector were transfected into GS cells, and cells were harvested at 24, 36, and 48 h. The transcription levels of host immune factors and pro-inflammatory cytokines were detected using qRT-PCR. As shown in Figure 7, expression levels of interferon related cytokines or effectors, including IRF3, IRF7, MDA5, ISG15, LGP2, MXI, IFP35, MyD88, and TRAF6, were all decreased in EcAtg5 overexpressing cells compared with control vector transfected cells. In addition, we also found that the expressions of pro-inflammatory cytokines such as interleukin (IL)-1β, IL-6, IL-8, and tumor necrosis factor alpha, were all significantly decreased in EcAtg5 overexpressing cells (Figure 8).

### EcAtg5 Suppressed ISRE and IFN and NF-κB Promoter Activities

**Figure 9:**
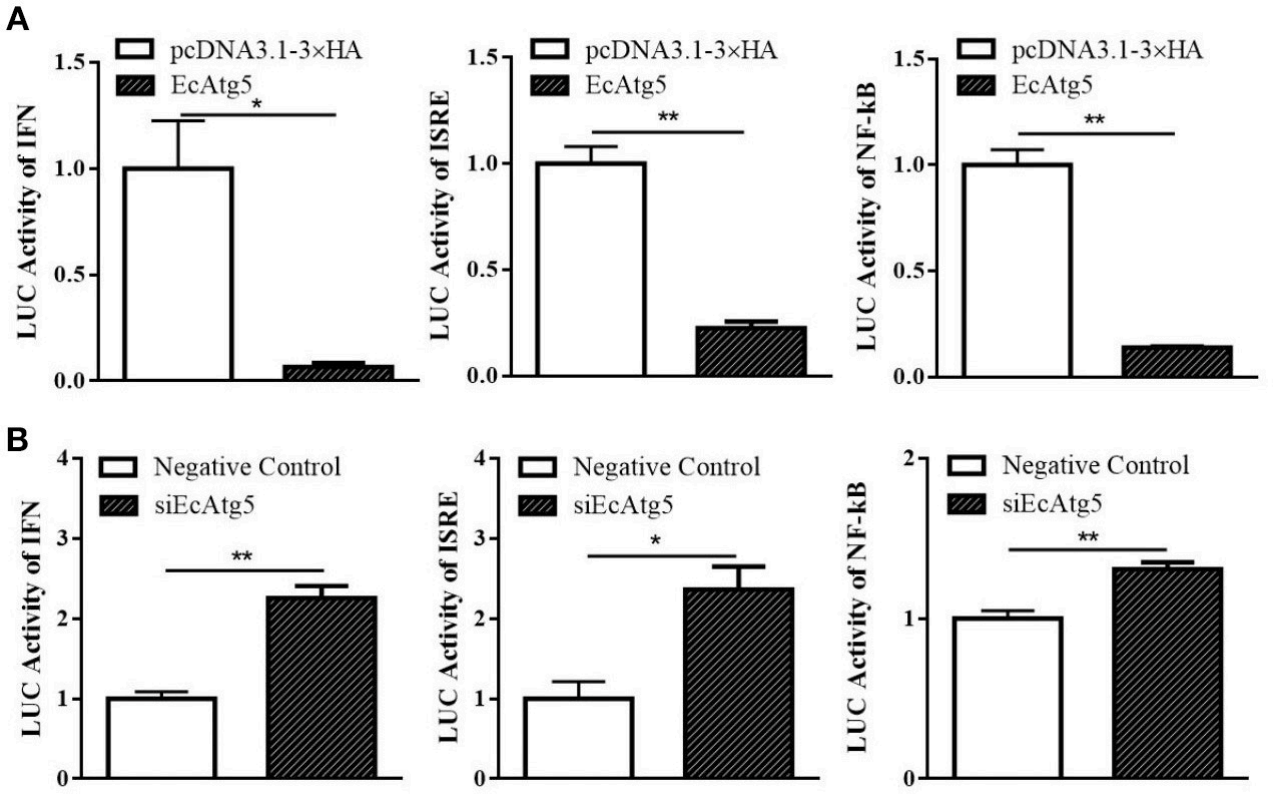
Effects of EcAtg5 on NF-κB response promoter, IFN promoter, and ISRE promoter activity. **(A)** Overexpression of EcAtg5 decreased promoter activities of NF-κB, IFN, and ISRE. **(B)** Knockdown of EcAtg5 increased promoter activities of NF-κB, IFN, and ISRE. The promoter activity was measured using the luciferase reporter gene assay. One-way ANOVA was used to evaluate the variability between treatment groups (*p < 0.05, **p < 0.01).

To further explore the roles of EcAtg5 during fish virus infection, the promoter activity of reporter genes in typical antiviral pathways, including ISRE, type I IFN, and NF-κB, were measured using the plasmids ISRE-Luc, INF-Luc, and NF-κB-Luc. As shown in Figure 9A, EcAtg5 overexpression suppressed the promoter activity of these genes. In addition, siEcAtg5 was co-transfected with the plasmids ISRE-Luc, INF-Luc, and NF-κB-Luc, and the promoter activity of three reporter genes were measured. The results showed that siRNA-mediated Atg5 knockdown increased the promoter activity of three reporter genes (Figure 9B). Thus, we proposed that EcAtg5 negatively regulates NF-κB and the IFN immune responses.

### Effects of EcAtg5 Overexpression on Cell Cycle Progression

**Figure 10:**
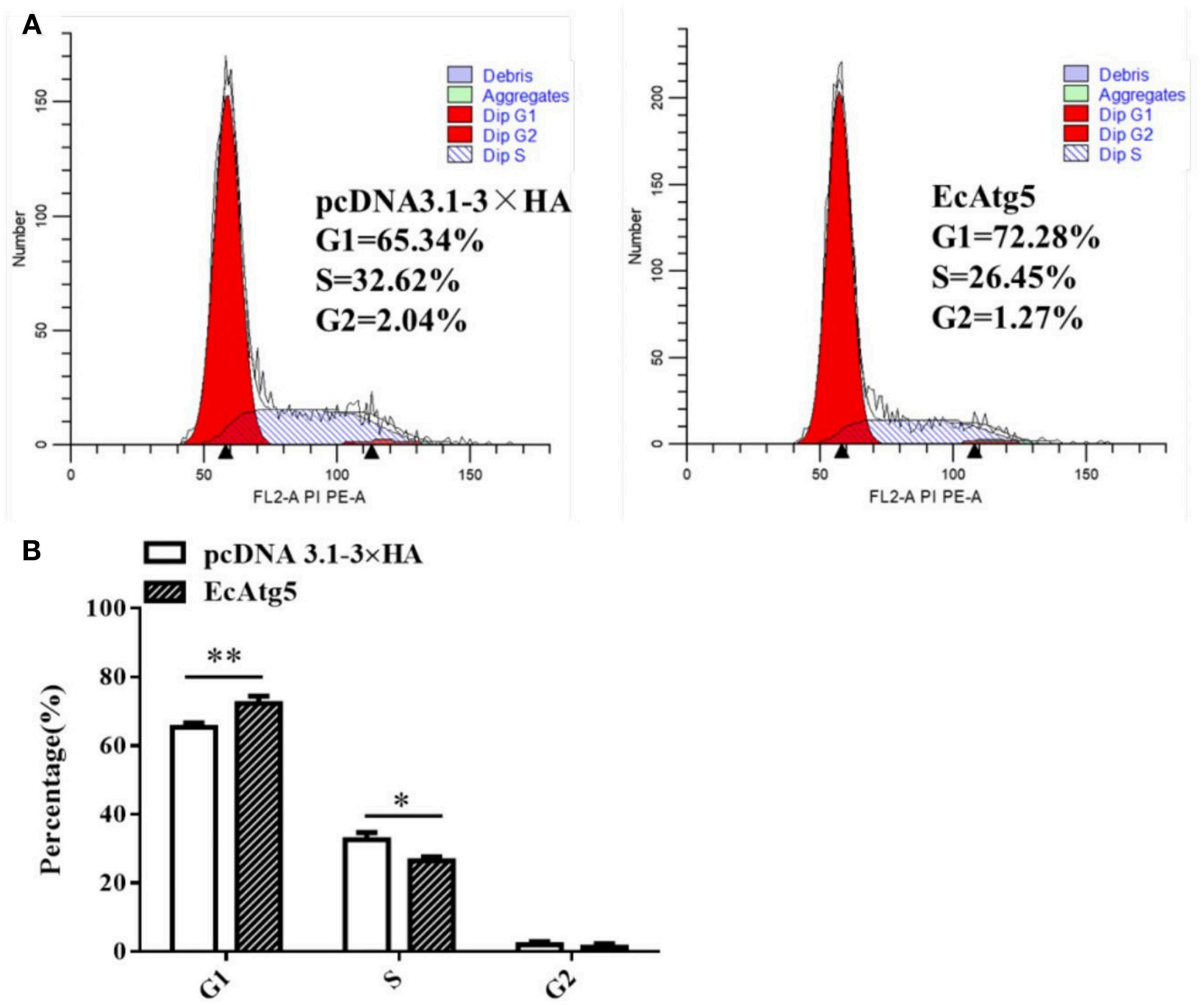
Effects of EcAtg5 overexpression on cell cycle progression. **(A)** GS cells (transfected with pcDNA3.1-3 × HA or EcAtg5) were harvested and analyzed using flow cytometry. **(B)** Quantitative analysis of the percentages of cells in different cell phases. One-way ANOVA was used to evaluate the variability between treatment groups (*p < 0.05, **p < 0.01).

Mammalian Atg5 plays a causal role in regulating cell cycle progression (26, 27). Whether EcAtg5 has the similar effects on cell cycle remains uncertain. To explore the role of EcAtg5 on cell cycle progression, GS cells were transfected with pcDNA3.1-3 × HA or EcAtg5. Ectopic expression of EcAtg5 clearly inhibited the G1/S transition compared to the empty vector overexpressing cells. The percentages of G1 phase cells in pcDNA3.1-3 × HA and EcAtg5 overexpressing cells were 65.34 and 72.28%, respectively (Figure 10). Those results indicated that EcAtg5 may affect cell cycle progression from the G1 to the S phase and arrest cells in the G1 phase.

## Discussion

Autophagy is a highly conserved pathway, and it plays an important role in resistance to intracellular viruses and other pathogens (28). Autophagosome formation relies on the Atg family (29), and Atg5 has been studied in detail in mammals. Atg5 is involved in autophagic membrane extension and curvature, LC3 (Atg8) recruitment, and lysosome and late intracellular regeneration. It also has been implicated in the IFN immune response, inflammation response, and lipid metabolism (25, 30). In fish, the roles of Atg5 gene in zebrafish neurogenesis and organogenesis has been reported, and the results showed that the formation of the Atg5-Atg12 conjugate may depend on Atg5 protein generation and its splicing (31). Twelve autophagy-related genes from yellow catfish *Pelteobagrus fulvidraco* and their transcriptional responses to waterborne zinc exposure were also characterized (32). However, little has been reported about the roles of Atg5 in virus replication and its relationship with the innate antiviral immune response in aquatic animals by far. In the present study, an Atg5 homolog from orange-spotted grouper (EcAtg5) was cloned and its roles during fish virus infection were investigated. EcAtg5 encoded a 275-amino acid protein that shared 94% and 81% identity to seabass (*Lates calcarifer*) and humans (*Homo sapiens*), respectively. Atg5 functions as an E3 ligase-like enzyme (33). EcAtg5 possesses all the characteristic features of canonical ubiquitin ligase, including two ubiquitin-like domains, a helix-rich domain, and the conserved calpain cleavage sites (32, 33).

As a preliminary step to unravel the physiological role of EcAtg5, the mRNA tissue distribution was determined. The present results indicated that the mRNA expression of EcAtg5 was ubiquitous within all the tested tissues. The ubiquitous distribution suggested that autophagy was implicated in many metabolic pathways among the tissues. Originally autophagy was identified as a response to nutrient deficiency, so it is thought to be a receptor of cellular energy and metabolism (34). However, it is now evident that autophagy can be induced by a variety of factors, including starvation, reactive oxygen species, endoplasmic reticulum stress, microbial invasion and so on (35). Based on this, we detected the expression of EcAtg5 under the stimulation of two viruses. Transcription levels of EcAtg5 increased from the early stage of RGNNV infection, suggesting that RGNNV infection may significantly induce autophagy activity to facilitate its proliferation. In SGIV infected cells, the expression levels of EcAtg5 firstly decreased within 24 h post-infection and then increased after 36 h. This pattern might be caused by the lack of cellular nutrition. With the cell growth, metabolism and virus replication, the nutrient deficiency in cells will increase autophagy activity over time.

Autophagy is an important cellular process by which Atg5 initiates the formation of double membrane vesicles (DMVs). Recently, the contribution of an autophagy protein, Atg5, to viral replication has been demonstrated (36), and Atg5 was identified as an interacting protein for the hepatitis C virus NS5B (37). The altered expression level of EcAtg5 in GS cells infected with SGIV and RGNNV suggested that EcAtg5 might play an essential role in the grouper response to fish virus infection, so the impact of EcAtg5 overexpression on virus proliferation were investigated. Overexpression of EcAtg5 promoted SGIV and RGNNV replication, evidenced by the severity of CPE, the increased transcription levels of viral genes, and the increased levels of viral proteins. Knockdown of EcAtg5 decreased SGIV and RGNNV replication by assessing transcription and protein levels of viral genes. The results suggested that Atg5 might share conserved function to viral replication from fish to mammals.

Studies of mammals suggested that Atg5-Atg12 promotes viral replication by negatively regulating the IFN response (38). The Atg5-Atg12 conjugate interacts directly with the mitochondrial antiviral-signaling protein (MAVS) and retinoic acid-inducible gene I (RIG-I) through the N-terminal caspase recruitment domain (CARD), resulting in inhibition of type I IFN production (38). N-terminal fragments of RIG-I and IFN-α possess great capacity to activate IFN-β and ISRE promoters in Atg5 deficient cells (39). When the formation of autophagosomes was promoted, the activity of the IFN-β promoter was decreased so that autophagy contributed to sustained hepatitis C virus infection (40). Here, overexpression of EcAtg5 in grouper cells not only decreased the expression levels of several interferon related cytokines or effectors, but also negatively regulated the expression of pro-inflammatory factors. Moreover, the ectopic expression of EcAtg5 significantly decreased ISRE, IFN, and NF-κB promoter activities, and knockdown of EcAtg5 raised promoter activities of these reporter genes. Atg5-Atg12 inhibits the production of IFN in canonical autophagy while playing the opposite role in alternative autophagy (41). Thus, overexpression of EcAtg5 might activate canonical autophagy in GS cells. Overexpression of EcAtg5 up-regulated the level of LC3-II, indicating that EcAtg5 can activate autophagy. Taken together, we speculated that EcAtg5 decreased interferon immune response and activated autophagy might contribute greatly to its promoting effect on SGIV and RGNNV replication.

In mammals, Atg5 can induce cell cycle arrest at the G1/S phase by up-regulating expression of p21 (a cyclin-dependent kinase inhibitor) at the level of post-transcription in response to challenges such as nutrient deficiency (42, 43). Considering that Atg5 is a key and relatively conserved protein, we speculated that fish Atg5 might play a similar role in cell cycle progression. In the present study, EcAtg5 affected cell cycle progression from the G1 to the S phase and arrested cells in the G1 phase. It was also reported that the replication level and virus titer of RGNNV were greater in cells released from the G1 phase or S phase of the cell cycle compared to cells released from the G2 phase (44). Those results suggested that overexpression of EcAtg5 may facilitate RGNNV replication. However, whether fish Atg5 affects the cell cycle by regulating p21 requires further investigation.

In conclusion, a key autophagy related gene (Atg5) from orange-spotted grouper (*E. coioides*) (EcAtg5) was cloned, and the roles of EcAtg5 in autophagy, innate immunity, and cell cycle were investigated in this study. The results showed that EcAtg5 plays crucial roles in virus replication via promoting autophagy, down-regulating antiviral IFN responses, and affecting cell cycle. This study identified a link between the autophagic machinery and innate immune signaling against viral infection.

## Data Availability

The datasets for this manuscript are not publicly available because this data has not been published. Requests to access the datasets should be directed to Jingguang Wei, weijg@scau.edu.cn.

## Ethics Statement

All animal-involving experiments of this study were approved by the Animal Care and Use Committee of College of Marine Sciences, South China Agricultural University, and all efforts were made to minimize suffering.

## Author Contributions

QQ and JW designed the experiments. CL performed the majority of the experiments, analyzed data, and wrote the manuscript. JL and XZ contributed experimental suggestions. SW, YH, and XH helped to design the experiments. All authors revised the manuscript.

### Conflict of Interest Statement

The authors declare that the research was conducted in the absence of any commercial or financial relationships that could be construed as a potential conflict of interest.
